# Biogenic Synthesis of Selenium and Copper Oxide Nanoparticles and Inhibitory Effect against Multi-Drug Resistant Biofilm-Forming Bacterial Pathogens

**DOI:** 10.3390/biomedicines12050994

**Published:** 2024-04-30

**Authors:** Rida Rasheed, Abhijnan Bhat, Baljit Singh, Furong Tian

**Affiliations:** 1 University of Wah, Wah Cantonment 47040, Pakistan; rida.rasheed268@gmail.com; 2 School of Food Science & Environmental Health, Technological University Dublin (TU Dublin), Grangegorman, D07 ADY7 Dublin, Ireland; d21126441@mytudublin.ie (A.B.); baljit.singh@tudublin.ie (B.S.); 3 MiCRA Biodiagnostics Technology Gateway and Health, Engineering & Materials Sciences (HEMS) Hub, Technological University Dublin (TU Dublin), D24 FKT9 Dublin, Ireland; 4 Nanolab Research Centre, FOCAS Research Institute, Technological University Dublin (TU Dublin), Camden Row, D08 CKP1 Dublin, Ireland

**Keywords:** antimicrobial resistance, bacterial pathogens, *E. coli*, *S. aureus*, biofilm, nanoparticles, MIC

## Abstract

Antimicrobial resistance (AMR), caused by microbial infections, has become a major contributor to morbid rates of mortality worldwide and a serious threat to public health. The exponential increase in resistant pathogen strains including *Staphylococcus aureus* (*S. aureus*) and *Escherichia coli* (*E. coli*) poses significant hurdles in the health sector due to their greater resistance to traditional treatments and medicines. Efforts to tackle infectious diseases caused by resistant microbes have prompted the development of novel antibacterial agents. Herein, we present selenium and copper oxide monometallic nanoparticles (Se-MMNPs and CuO-MMNPs), characterized using various techniques and evaluated for their antibacterial potential via disc diffusion, determination of minimum inhibitory concentration (MIC), antibiofilm, and killing kinetic action. Dynamic light scattering (DLS), scanning electron microscopy (SEM/EDX), and X-ray diffraction (XRD) techniques confirmed the size-distribution, spherical-shape, stability, elemental composition, and structural aspects of the synthesized nanoparticles. The MIC values of Se-MMNPs and CuO-MMNPs against *S. aureus* and *E. coli* were determined to be 125 μg/mL and 100 μg/mL, respectively. Time–kill kinetics studies revealed that CuO-MMNPs efficiently mitigate the growth of *S. aureus* and *E. coli* within 3 and 3.5 h while Se-MMNPs took 4 and 5 h, respectively. Moreover, CuO-MMNPs demonstrated better inhibition compared to Se-MMNPs. Overall, the proposed materials exhibited promising antibacterial activity against *S. aureus* and *E. coli* pathogens.

## 1. Introduction

Nanotechnology has established itself as a dynamic and interdisciplinary branch of research in recent years [1,2,3]. Nanomaterials have a wide range of physicochemical properties that make them useful in many cutting-edge fields and have played an immense influence on the industrial revolution, which has led to the establishment of hundreds of innovative products [4]. The domains of health, agriculture, and other fields all have intriguing applications for metal nanoparticles [5]. The emergence of antibiotic-resistant microorganisms is one of the biggest risks to public health. Antimicrobial resistance has been identified by the World Health Organization as one of the major global health issues which has resulted in higher rates of morbidity and mortality among patients [6]. Antimicrobial resistance (AMR) was a contributing factor in 4.95 million deaths in 2019, of which resistant infections were the direct cause of 1.3 million deaths [7]. Several factors contribute to the emergence of AMR, including genetic mutations; incomplete antibiotic treatment, which allow pathogens to survive and develop resistance; as well as the overuse and misuse of antibiotics [8]. Biofilms also create the ideal environment for bacterial colonization and growth and the emergence of potentially fatal microbial infections [9]. These biofilms not only significantly contribute to the progression of diseases but also pose a formidable challenge in treatment efficacy. They create a highly protective environment that shields bacteria from major external stressors including the host’s immune response and antibiotics, thus limiting the effectiveness of available treatments [7]. Antibiotic-resistant and infectious diseases caused by these pathogens are one of the most significant health-related concerns of the 21st century [10]. Therefore, the challenge of restoring susceptibility to antimicrobial resistance and the urgent imperative to curb bacterial infections in healthcare have placed a heavy responsibility on researchers to innovate and bring forth novel antimicrobial treatments capable of combating the threat. Nevertheless, there is a looming concern that the pace of antibiotic resistance (as these contagions tenaciously continue to develop adaptable defenses against pre-existing treatments [11]) outpaces the development of novel treatments. This underscores the necessity for a deeper comprehension of the molecular and ecological factors dictating the propagation of antibiotic resistance. Consequently, researchers can be better equipped to fulfill the demand for novel compounds having antibacterial potential including potent agents that can inhibit biofilm formation.

The synthesis and assembly of new antibiotics are labor-intensive and expensive, as well as time-consuming. Therefore, it has become highly endorsed to develop unusual, alternative methods to treat infectious diseases [12]. The pharmaceutical and biomedical industries are currently addressing the problem of an ongoing rise in infectious agents that are antibiotic-resistant, as well as the intimidation of emerging and re-emerging multi-drug-resistant pathogens. Thus, in the current scenario, the amalgamation and fabrication of antimicrobial compounds to increase antimicrobial capability constitute the priority fields of research [13]. To combat microbial resistance using metal nanoparticles (MNPs) holds considerable potential. A broad spectrum of Gram-positive and Gram-negative bacteria have been shown to be susceptible to the antibacterial effects of metallic nanoparticles [14,15,16]. Furthermore, metal oxide nanoparticles (MONPs) with highly effective antimicrobial properties against a variety of pathogens such as *Pseudomonas aeruginosa*, *Klebsiella pneumonia*, and *E. coli* includes Ag_2_O, ZnO, Au, TiO_2_, and ZrO_2_ nanoparticles [17,18]. Compared to traditional antibiotics, nanoparticles offer numerous benefits which include improved precision in targeting infected tissues, extended duration of antibiotic activity, greater solubility, increased stability, enhanced ability to penetrate epithelial barriers, and minimized risk of side effects [19]. Furthermore, a significant number of nanoparticles have natural antibacterial qualities that help fight bacterial infections. These include preventing the development of biofilms, increasing the production of reactive oxygen, and damaging bacterial cell membranes, proteins, and DNA [20].

Se and CuO monometallic nanoparticles have drawn a significant amount of attention from researchers and have been used in a variety of biological applications [21,22], such as antifungal [23], drug delivery, antioxidant, antibacterial [24], and anticancer activities [23]. The strong biological activity and low toxicity of the Se-MMNPs suggest that they have the potential to combat infections caused by Gram-negative and Gram-positive microorganisms [25]. According to the literature, selenium nanoparticles interfere with the bacterial genetic material as well as disrupt the microbial membrane [26]. Various methods are used for the production of nanoparticles, for example, physical and chemical methods, but these are costly and harsh to the surrounding environment and living entities. Furthermore, these methods require high expertise for nanoparticle production [27]. Therefore, an effective and appropriate method is a prerequisite for nanoparticle production. Over recent years, green nanotechnology has gained considerable attention in many fields of nanoscience and nanomedicines. Green synthesis suggests a clean, non-hazardous, and environmentally friendly approach with a varied assortment of properties such as size, shape, and composition. This eco-friendly approach is more beneficial and valuable and tends to be faster, cost-effective, and easy [28].

Many fungi like *Trichoderma harzianum* [29], *Penicillium chrysogenum* [23], *Aspergillus niger* [30], and *Aspergillus fumigatus* [31] have been used with outstanding results for the synthesis of various nanoparticles. The literature demonstrates that fungi are the most effective biological agents for producing metal nanoparticles among the various biological agents [13]. The fungus-mediated synthesis of nanoparticles offers numerous benefits, such as easy and simple scaling up, economic feasibility, processing of biomass, non-hazardous, and high yield [32,33]. The genus *Talaromyces*, due to its ability to secrete a variety of intriguing secondary metabolites, has received a significant amount of attention [34]. *Talaromyces* species are normally soil-based and secondary metabolites produced by the *Talaromyces* demonstrate auspicious anti-cancer, anti-microbial, and anti-proliferative activities [35].

In this study, Se-MMNPs and CuO-MMNPs were synthesized and well characterized using UV–Vis, DLS, XRD, and SEM-EDX analyses. The antimicrobial activity of synthesized nanoparticles was examined against *Escherichia coli* (*E. coli*) and *Staphylococcus aureus* (*S. aureus*). This work presents an easy, green, and scalable method for the synthesis of Se-MMNPs and CuO-MMNPs as potential candidates against pathogens and AMR development. To the best of our knowledge, this is the first time *Talaromyces haitouensis* extract has been used to synthesize Se-MMNPs and CuO-MMNPs.

## 2. Materials and Methods

### 2.1. Chemicals and Reagents

All the chemicals of analytical grade including CuSO_4_·5H_2_O and Na_2_SeO_3_, Muller Hinton Agar (MHA), Muller Hinton Broth (MHB), Sabouraud Dextrose agar (SDA), and Sabouraud Dextrose Broth (SDB) were purchased from Sigma-Aldrich. The bacterial strains *E. coli* (692642) and *S. aureus* (668830) used in the study were obtained from the Microbiology Diagnostic Lab, Islamabad, Pakistan. These strains were resistant to multiple antibiotics; *E. coli* was resistant to Minocycline, Gentamicin, Imipenem, and Doxycycline; *S. aureus* was resistant to Ciprofloxacin, Levofloxacin, Erythromycin, Azithromycin vancomycin, etc. The bacterial strains were cultured in Mueller Hinton Broth (MHB) at 37 °C for 24 h. Double-distilled water (DDW) and 70% ethanol were used throughout the experiments.

### 2.2. Synthesis of Monometallic Nanoparticles

Monometallic nanoparticles were synthesized via slight modifications of the procedures used by Wang et al. [1]. An aqueous solution of CuSO_4_·5H_2_O and Na_2_SeO_3_ were combined with 25 mL of cell-free culture filtrate (CFCF) gradually. The mixture was then incubated at ambient temperature. To diminish the possibility of photo-oxidation, flasks were kept covered with aluminum foil and put under magnetic stirring for 24 h at 45 °C. The time limits were established in accordance with the standardization and were subsequently validated using the various methods outlined below. The time limits were finalized to correlate to the color change. After cooling at room temperature, the reaction was centrifuged at 14,000 rpm. To obtain the purified nanoparticles, the pellet was then washed with 70% ethanol and lyophilized (FreeZone 6, Labconco, Kansas City, MO, USA) for 24 h. The synthesized copper oxide and selenium monometallic nanoparticles were then kept at ambient temperature and under darkness in polypropylene tubes [36].

### 2.3. Physiochemical Characterization of Se and CuO Monometallic Nanoparticles

The synthesized Se-MMNPs and CuO-MMNPs were characterized using various spectroscopic techniques. The copper oxide and selenium monometallic nanoparticles (MMNPs) were confirmed by the UV–vis spectrum. Using a UV–vis spectrophotometer (U-2900 UV–vis Spectrophotometer—HITACHI High-Tech Science, Tokyo, Japan; λ = 200–1100 nm) and distilled water as the reference solution, the absorbance of synthesized MMNPs was measured over the wavelength range of 200–800 nm [37]. According to the previously reported method, dynamic light scattering (DLS; Microtrac Nanotrac Wave II, York, PA, USA) and zeta potential studies were carried out using a Zeta sizer Nano ZS (Malvern, Malvern Hills, UK) [38]. X-ray diffraction (XRD) of the dried powder sample of monometallic nanoparticles was carried out to analyze the crystallinity and phase purity of the sample using D8 Advance Bruker to calculate broad-angle X-ray diffractograms at 60 kv and 60 mA current. Scanning electron microscopy (SEM, Hitachi S-3000N, Tokyo, Japan) was used to characterize the morphology and size of the purified Se-MMNPs and CuO-MMNPs [39,40].

### 2.4. Antibacterial Activity

The disc diffusion method was utilized to evaluate the bactericidal activity of Se-MMNPs and CuO-MMNPs separately against the two bacterial strains on the MHA medium. As test organisms, *S. aureus* (Gram-positive) and *E. coli* (Gram-negative) were used. The bacterial strains (1.5 × 10^8^ CFU/mL) were initially cultured on MHA medium and uniformly distributed using a sterile spreader. The discs were prepared with the Se-MMNPs and CuO-MMNPs and placed on the MHA plates. Discs prepared with dimethyl sulfoxide (DMSO) (2%, *v*/*v*) were used as a control. The diameter of the inhibitory zone was gauged after the plates were incubated for 24 h at 37 °C. There were three measurements for each experiment [41].

### 2.5. Determination of Minimum Inhibitory Concentration (MIC)

The micro broth dilution method was performed to ascertain the minimal inhibitory concentration (MIC) of the synthesized monometallic nanoparticles against antimicrobial-resistant strains (*S. aureus* and *E. coli*). MHB having different NP concentrations (100–500 μg/mL) was added to each microplate well and placed on an orbital shaker (Heidolph Instruments, Schwabach, Germany) at 37 °C for 24 h. The next step was to fill each well with 50 μL of bacterial suspension. The pure bacterial suspensions (devoid of nanoparticles) and MHB (devoid of any bacterial suspension) served as the positive and negative controls. A microplate reader (Thermo, Multiskan Go, Waltham, MA, USA) was used to measure the absorbance at 600 nm, and the findings were recorded. Finally, the MIC was entrenched as the lowest concentration of Se and CuO-MMNPs hindering the target pathogenic bacteria. All experiments were carried out under necessary protocols and the tests were carried out in triplicates [42].

### 2.6. Killing Kinetics Study

The synthesized nanoparticles were subjected to the killing kinetics method as already reported [43]. In a nutshell, overnight colonies of each bacterial strain were re-cultured in MHB and incubated (2 h, 37 °C). The MIC concentrations of Se and CuO-MMNPs were added to MHB media with bacterial inoculum (1 × 10^6^ CFU/mL) and the mixture was subsequently incubated at 37 °C. The bacterial suspension without being subjected to any nanoparticles was used as a control. Bacterial cell viability was determined at different intervals.

### 2.7. Inhibition of Biofilm Formation

The crystal violet 96-well micro titer plate (MTP) assay was used to verify the antibiofilm efficacy of Se and CuO-MMNPs with nominal modifications to prevent or minimize the biofilm aggregation of clinical pathogens. In brief, MIC values of Se and CuO-MMNPs were added to an overnight 50 µL bacterial suspension of *S. aureus* and *E. coli* (robust biofilm-producing strain) in 200 µL of culture medium and incubated at 37 °C for 48 h. The cells devoid of any nanoparticles were used as a control. The wells were washed with phosphate-buffered saline (PBS) (pH 7.4) after the 24 h incubation. The adhered biofilm for 15 min was subjected to 0.1% crystal violet and then gently cleaned with PBS. The optical density of the pigmented biofilm was calculated to be 595 nm after it was dissolved in 200 μL of 95% ethyl alcohol. Eventually, crystal violet confined biofilm was assessed using a cell imager (Evos^®^R FL Cell Imaging System; Thermo Fisher Scientific, Waltham, MA, USA). The Se-MMNPs and CuO-MMNPs were treated and untreated results were compared [44].

### 2.8. Statistical Analysis

The data from the MIC values of the Se-MMNPs and CuO-MMNPs were analyzed with Graph Pad Prism version 8.4.2. The results are shown as the mean ± standard deviation (SD) of three independent replicates (*p* < 0.05).

## 3. Results and Discussion

### 3.1. Biogenic Synthesis of Se-MMNPs and CuO-Monometallic Nanoparticles

**Scheme 1:**
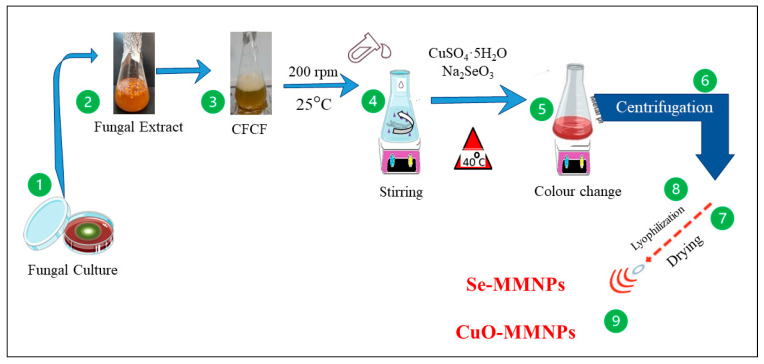
Schematic representation of synthesized Se-MMNPs and CuO-MMNPs.

*Talaromyces haitouensis* CFCF was employed for the synthesis of Se-MMNPs and CuO-MMNPs, as shown in Scheme 1.

The method was adopted from the literature [39]. The cell-free culture filtrate was added to the 1 mM solution of CuSO_4_·5H_2_O and Na_2_SeO_3_ individually for the synthesis of monometallic nanoparticles and then the reaction continued in the dark condition at ambient temperature (Step 3 Scheme 1). The reduction in sodium selenite and copper ions in cell-free culture filtrate resulted in noticeable Se-MMNPs and CuO-MMNPs synthesis, which was observable as a change in the color of the solution throughout the incubation time (Step 5 in Scheme 1). A light blue color was observed in the extract after the addition of CuSO_4_·5H_2_O solution [45]. After 24 h of reaction, the reaction mixture was analyzed by UV–vis (Step 9 in Scheme 1). Then, the synthesized NPs were collected through centrifugation at 14,000 rpm and stored in a dark condition at room temperature for further characterization and bioassay [46]. Similarly, a color from white to a brick red was observed in extract after the addition of Na_2_SeO_3_, in which Se^+4^ was reduced to Se^0^ after the same conditions mentioned above [45]. *Talaromyces haitouensis* extract has the ability to produce two different kinds of monometallic nanoparticles under the same conditions.

### 3.2. Characterization of Synthesized Monometallic Nanoparticles

**Figure 1:**
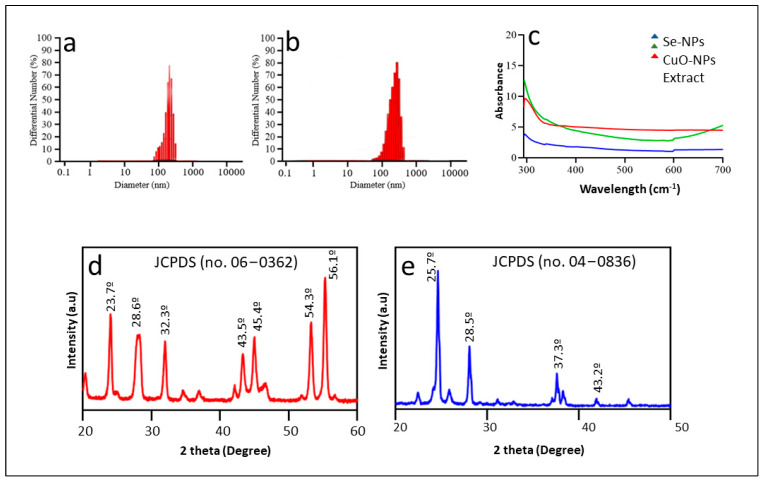
(**a**,**b**) Dynamic light scattering (DLS) of Se-MMNPs and CuO-MMNPs, (**c**) UV−vis absorption spectra of monometallic NPs, and (**d**,**e**) XRD patterns for Se-MMNPs and CuO-MMNPs.

The characteristic of Se-MMNPs and CuO-MMNPs was confirmed by spectroscopic techniques. The dynamic light scattering analysis of the appropriately distributed Se-MMNPs in distilled water gauged that the nanoparticles had an average diameter of 150 nm (Figure 1a). The DLS study revealed that the polydispersity index for CuO-MMNPs was 0.28 and the average particle size was 200 nm (Figure 1b). All the biological activities of the monometallic nanoparticles are strongly affected by the nanoparticles’ size as the diameter of the nanoparticles is a highly significant defining property. The Se-MMNPs had a zeta potential of 200 mV, which elucidates the strong repulsion between the particles and thus escalates the stability of the nanoparticles [47]. According to Mali et al. (2020), *Celastrus paniculatus* extract-mediated Cu-NPs had an average particle size of 290 nm and a polydispersity index of 1.00 [48]. It has also been observed that *Artabotrys odoratissimus* leaf extract can also synthesize Cu-NPs between 115 and 135 nm [49].

UV–vis spectroscopy at a wavelength ranging from 300 to 700 nm was ascertained for the surface Plasmon response (SPR) of the selenium colloidal solution. During the synthesis of Se-MMNPs, the characteristic SPR was observed and a peak was found between 200 and 400 nm [26]. The synthesis of CuO-MMNPs was first monitored by the color changes followed by UV–vis spectroscopy. A peak at 330 nm was observed (Figure 1c) due to the surface plasmon resonance (SPR). The SPR at 330 nm shows the synthesis of CuO-MMNPs, and similar spectra have been reported in the literature showing the Cu-NPs’ absorption band at 326 nm using leaf extract of *Ageratum houstonianum* [49].

The XRD-diffraction peaks were observed at 2θ values of 23.78, 28.6, 32.3, 43.5, 45.4, and 56.1°, which correspond to the Bragg’s reflections at (100), (101), (110), (200), (201), and (210), showing the crystalline nature of Se-MMNPs, which were in line with JCPDS (Figure 1d) [50]. XRD analysis was used to confirm the crystalline structure of CuO-MMNPs (Figure 1e). CuO diffraction peaks were observed at 2θ with 32.1, 35.1, 38.2, 48.6, and 52.3°, and these patterns corresponded to (110), (002), (111), (202), and (020), respectively. The peaks of all the CuO-MMNPs with a standard card were similar to those of the Joint Committee on Powder Diffraction Standards, as shown in Figure 1e. Hence, the findings explicitly validate the synthesis of CuO-MMNPs [43]. Debye Scherrer’s equation was used to determine the average crystallite size of Se-MMNPs and CuO-MMNPs, which were ~150 nm.

**Figure 2:**
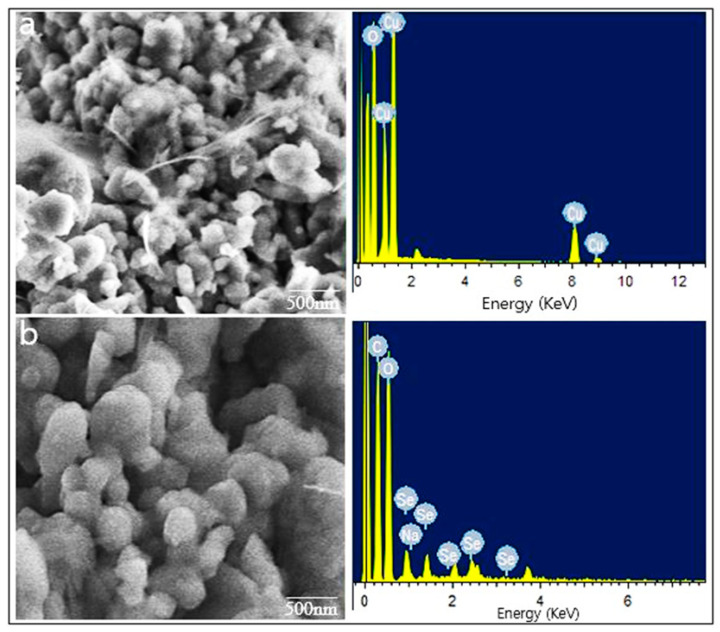
Left (**a**,**b**): SEM images of Se-MMNPs and CuO-MMNPs. Right: EDX spectra confirming the presence of Se and CuO in the synthesized nanoparticles.

EDX analysis of synthesized nanoparticles demonstrated the intensity, phase purifications, and crystallinity of Se-MMNPs and CuO-MMNPs. The crystal structure and phase of the synthesized Se-MMNPs and CuO-MMNPs were examined using SEM and EDX analysis in Figure 2.

SEM coupled with the EDX was recorded to visualize the texture and diameter of Se-MMNPs and CuO-MMNPs. The crystalline spherical-shaped metallic nanoparticles are shown in the SEM images (Figure 2). Se-MMNPs with spherical agglomerated morphology and a size range of 120–200 nm were observed in accordance with the already reported results [51]. EDX analysis revealed the presence of selenium and carbon components, as shown in Figure 2a. Similarly, CuO-MMNPs demonstrated a spherical smooth shape with a size ranging from 100 to 150 nm, as shown in Figure 2b. These trends in SEM are in agreement with the XRD analysis and previously reported literature [52].

### 3.3. Qualitative Antimicrobial Assays

**Figure 3:**
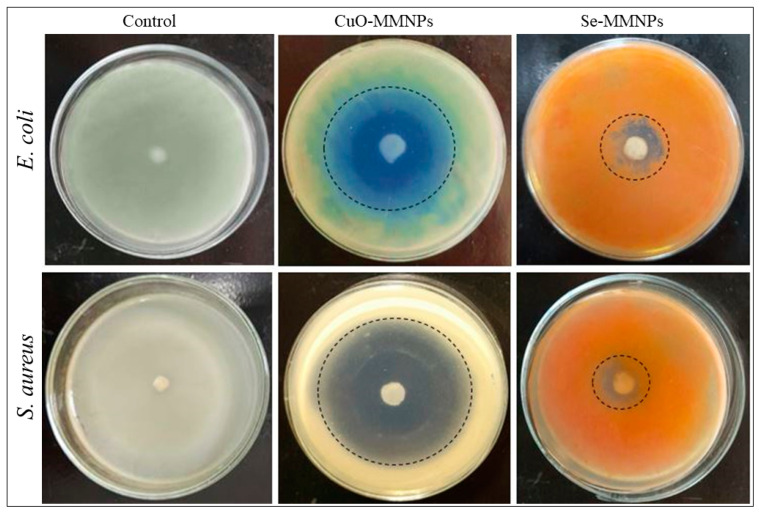
Zone of inhibition of Se-MMNPs and CuO-MMNPs against multidrug-resistant *E. coli* and *S. aureus* strains.

The nanoparticles synthesized from the *Talaromyces haitouensis* secondary metabolites exhibited good in vitro antimicrobial activities, as demonstrated in Figure 3. Both Se-MMNPs and CuO-MMNPs caused a significant reduction in the growth of *S. aureus* and *E. coli*. Under Se-MMNPs treatment, the zone of inhibition against *S. aureus* was 19 ± 0.5 mm (Figure 3). On the other hand, the zone of inhibition against *E. coli* was 21 ± 0.3 mm. Moreover, under CuO-MMNPs treatment, a strong inhibitory effect was observed. The inhibition zone was 33 ± 0.3 mm and 30 ± 0.5 mm against *S. aureus* and *E. coli*, respectively. The disc diffusion test [53] revealed that the CuO-MMNPs had strong inhibition zones against *S. aureus* (33 ± 0.3 mm) and *E. coli* (30 ± 0.5 mm) surrounding them, indicating that they show a potential antibacterial activity. The experiment was carried out in triplicates.

**Figure 4:**
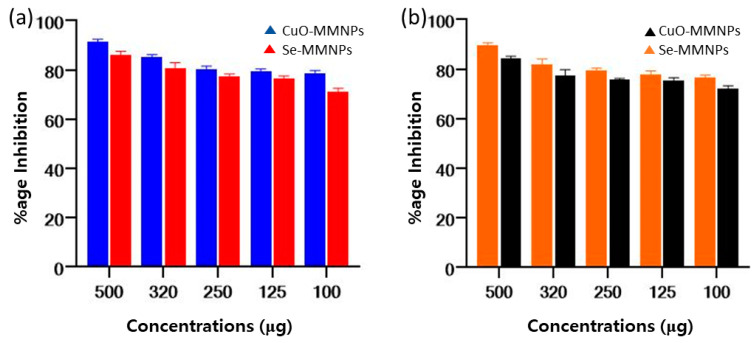
Growth percentage inhibition of *E. coli* (**a**) and *S. aureus* (**b**).

The MIC of Se-MMNPs and CuO-MMNPs for the aforementioned microorganism was also established, as depicted in Figure 4. These trends in the MIC data are consistent with findings from earlier research [52,54]. Smaller nanoparticles frequently have a large surface area, which makes it easier for them to contact the bacterial cell membrane and may eventually alter fundamental processes like penetrability and cell respiration, which can result in cell death [55]. The MIC value of Se-MMNPs and CuO-MMNPs from different concentrations (500, 320, 250, 125, and 100 μg/mL) were confirmed after the bacterial suspension was inoculated and incubated with antibacterial agents for 24 hrs. As a result, the MIC of Se-MMNPs was observed to be 125 μg/mL for *S. aureus* and *E. coli* and then the MIC concentration of CuO-MMNPs was observed to be 100 μg/mL for *S. aureus* and *E. coli*, showing that it has potent bacteriostatic action. These findings are consistent with the earlier research (Shende et al.) and demonstrate that metal nanoparticles exhibit promising antibacterial activities to combat microbial infections [56]. Srivastava et al. also reported that the *Ralstonia eutropha* biomass-mediated Se-NPs exhibited strong antibacterial activity against *P. aeruginosa*, *S. pyogenes*, and *A. clavatus* [57]. Furthermore, the MIC value of Cu-NPs used in this study is lower than that reported by Zain et al. against *Bacillus subtilis* and *E. coli* [58]. Nieto-Maldonado et al. reported that Gram-negative (*E. coli*) bacteria need a higher concentration of Cu-NPs than Gram-positive (*S. aureus*) bacteria. In comparison, our results show that CuO-MMNPs are more effective against *E. coli* (Gram-negative) regarding the MIC value [59]. The antibacterial results show that *S. aureus* and *E. coli* need a higher concentration of Se-MMNPs compared to CuO-MMNPs. Overall, our results show that the Se-MMNPs and CuO-MMNPs prevented the growth of selected bacterial strains. However, CuO-MMNPs exhibited strong antibacterial action as compared to Se-MMNPs.

### 3.4. Time–Kill Kinetics Assay

**Figure 5:**
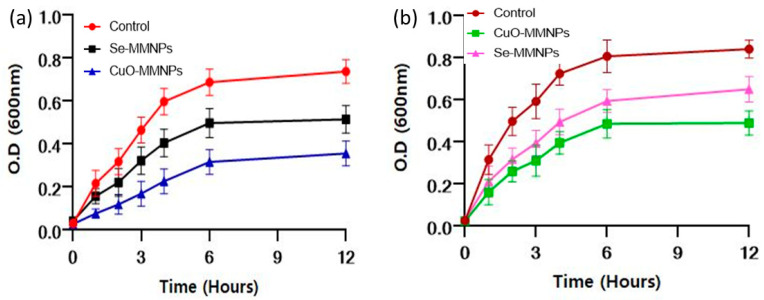
Time-dependent killing kinetic assays of Se-MMNPs and CuO-MMNPs against (**a**) *E. coli* and (**b**) *S. aureus*. The corresponding MIC concentration was used to measure the time efficacy.

The time–kill test was performed to observe the bactericidal action of Se-MMNPs and CuO-MMNPs. The monometallic nanoparticles at MIC were combined with an inoculum containing *S. aureus* and *E. coli* (1.5 × 10^8^ CFU/mL). According to the literature, exposing selected bacterial strains to nanoparticles for around 6 h maximizes the killing kinetic action [60]. A spectrophotometer was used to assess the absorbance of bacterial growth treated with MIC values at various time intervals, whereas the positive control was without any nanoparticles. In this study, the CuO-MMNPs-treated *S. aureus* growth was reduced after 3 h, while the growth of *E. coli* cells decreased after 3.5 h (Figure 5a,b). Similarly, Se-MMNPs-treated *E. coli* started diminishing after 5 h, followed by *S. aureus* after 4 h of incubation, as demonstrated in Figure 5a,b.

### 3.5. Quantitative Detection of Biofilm Formation

**Figure 6:**
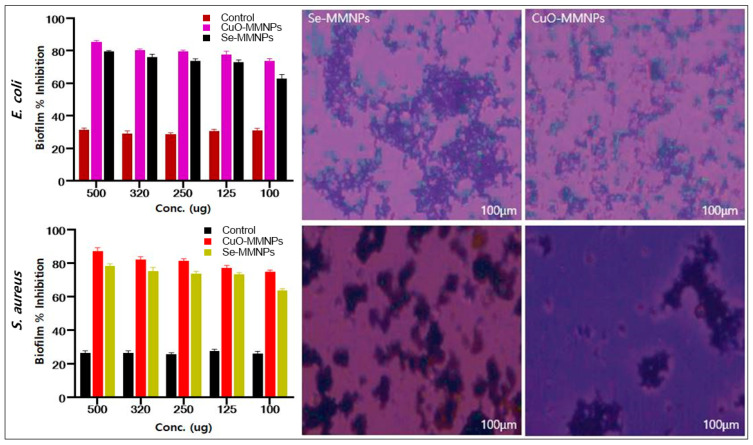
Determination of anti-biofilm activities of Se-MMNPs and CuO-MMNPs; images are labeled accordingly.

The efficiency of Se-MMNPs and CuO-MMNPs against selected strains in inhibiting biofilm development at the MIC concentration was evaluated in Figure 6.

Figure 6 shows that both Se-MMNPs and CuO-MMNPs can limit the formation of biofilms. According to the literature, bacterial biofilms are associated with antibiotic-resistant infections and are difficult to treat [61]. Our findings show that *S. aureus* and *E. coli* biofilms are successfully diminished by Se-MMNPs and CuO-MMNPs (*p* < 0.05). The suppression of biofilm and nanoparticle concentrations was found to be precisely proportional, indicating a dose-dependent action. Hence, Se-MMNPs and CuO-MMNPs were found to be effective biofilm disruptors. The experiment was performed in triplicates, and the photographs were analyzed using a cell imager.

### 3.6. Antibacterial Mechanism: Action of Nanomaterials and Unveiling the Battle against Bacterial Infections

**Scheme 2:**
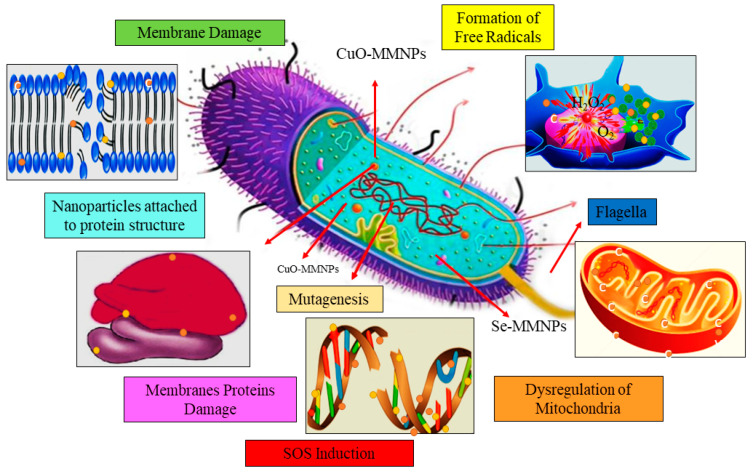
Schematic representation of multiple possible pathways involved in the antibacterial actions of various nanoparticles and in triggering the bacterial cell death.

The global quest for novel antimicrobial magic bullets is accelerating and becoming increasingly important as conventional antibiotics and antibacterial treatments fail to eradicate resistant bacteria and biofilms. Several studies have shown that nanomaterials could be useful in combating antimicrobial antibiotic resistance [62]. Direct association with bacterial cell walls, biofilm mitigation, and the production of reactive oxygen species are merely a few aspects [63]. Nanomaterials are more effective in treating antibiotic-resistant bacteria because they are capable of targeting these pathogens in a variety of ways, as depicted in Scheme 2 [20,64].

## 4. Conclusions

The synthesized monometallic nanoparticles were characterized by using various techniques and their inhibitory effect was tested against *E. coli* and *S. aureus.* DLS and SEM studies revealed that the particle size of the Se-MMNPs and CuO-MMNPs ranged from ~150 nm to ~100 nm. EDX analysis confirmed the presence of Se and Cu in the nanoparticles. The Se-MMNPs and CuO-MMNPs exhibited substantial antibacterial activity, with MIC values ranging from 100 to 500 μg/mL, against multi-drug resistant *E. coli* and *S. aureus* strains. The findings of killing kinetic action and anti-biofilm formation suggest that these materials could be potential candidates for combating bacteria, particularly against biofilm-forming strains. The sensitivity of Se-MMNPs and CuO-MMNPs as antimicrobial agents increases with nanoparticle concentrations while CuO-MMNPs showed better antibacterial activity than Se-MMNPs against *E. coli* and *S. aureus*. The studies on antibacterial, MIC, antibiofilm, and time–kill assays highlights the importance of CuO-based nanoparticles in combating *E. coli* and *S. aureus*. Overall, materials exhibit promising antibacterial activity, but further investigations (including biocompatibility efficacy) are necessary against a variety of other microbes to justify their significance in real-world applications.

## Data Availability

The data presented in this study are available on request from the corresponding author.
